# Supplementary material for: A UMLS-based spell checker for natural language processing in vaccine safety
Source: BMC Med Inform Decis Mak. 2007 Feb 12;7:3. doi: 10.1186/1472-6947-7-3 (PMC1805499; doi:10.1186/1472-6947-7-3)
Supplement: Additional file 13 — RAP application directory. Sets up directory for RAP (RDF application for PHP) source code [file 1472-6947-7-3-S13.gz › rap/doc/implNotes/database_backend.htm]

Implementation Notes: Database Backend


### RDF API for PHP V0.9.1

# Implementation Notes: Database Backend

  

Radoslaw Oldakowski <radol@gmx.de>
  
October 2003

This document is meant for developers who are interested in further work on RDF API for PHP.
I will disclose here some implementation issues of the new developed
Database Backend for RAP V0.6. However, my intention is not to describe each
method one by one but to explain some general concepts instead.

### Database Schema

One of the most fundamental decisions I was faced with as the developer of
the database backend for RAP was the choice of a suitable database schema. Among
various RDF APIs, there are different approaches to storing RDF data in a relational
database stretching from pain layouts, where statements are stored in one table
in a form resembling N-Triples, to highly normalized schemas consisting of several
tables enabling efficient implementation of sophisticated RDF capabilities (cf.
[Beckett
2003], [Melnik 2001]).

The goal for RAP's database backend was to provide an optimized for speed,
portable solution that would support all RAP's features and fitt into its internal
structure. Accordingly, the database layout should reflect all these requirements
as well.

At the beginning I designed a normalized database schema with the main table
*rdf\_statements* which stored only numerical identifiers pointing to the
corresponding entries in tables: *rdf\_models*, *rdf\_resources*, and
*rdf\_literals*. For this database layout I further developed a prototype
of RAP's database backend. Although this solution met all requirements of a
proper normalization, in practice it turned out to be definitely too slow. As
the result of that experience I developed another prototype, this time, using
a denormalized database schema, where all resources and literals were written
in full in table *statements*. Subsequently, to compare the performance
of both solutions, I did some benchmark tests with two databases: MsAccess 2000
and MySQL 3.23. These tests resulted in a finding that the simple model was
2-3 times faster than the explicit model and the advantage was rising with the
increasing number of statements. Moreover, the trade-off between better performance
and increased database size turned out to be acceptable, especially since RAP
mostly targets manipulating medium-sized RDF models (up to 100.000 statements).
After some minor changes and improvements (e.g. joint indexing on subject and
predicate) the denormalized layout has become the final RAP's database schema.
Its description can be found in the database
schema provided with the API documentation.

### Internal Structure of RAP's Database Backend

The core of RAP's database backend is built by two classes: `DbStore`
and `DbModel`. The former is used to create, store, list, and retrieve
persited models, whereas the latter provides methods for manipulating these
models. Furthermore RAP V0.8 utilizes ADOdb
Library for PHP V4.52 to connect to multiple databases in a portable manner.

Incorporating of the newly developed classes into RDF API for PHP entailed
a reorganization of RAP's class tree (i.e. renaming of the former class `Model`
to `MemModel` and creating of the parent class `Model`)
and moreover required a customization of numerous existing methods. Consequently,
the constructor of class `BlankNode` was made able to generate unique
identifiers for persisted models as well, and several methods of class `MemModel`
like: `containsAny()`, `containsAll()`, `equals()`,
`unite()`, `subtract()`, `intersect()`, `addModel()`
were adapted for receiving both MemModels and DbModels as a parameter.

In order to work with persisted models, at first, an instance of class `DbStore`
representing the database has to be created. Given the passed parameters, the
constructor method creates an `ADOConnection` object which performs
the connection to the database and is responsible for executing SQL statements.
This object is represented by the class internal variable `$dbConn`.

When a method `getModel()` or `getNewModel()` is called
on a database object, an instance of `DbModel` is returned. Every
object representing a persisted model stores four variables. The first two:
`$modelURI` and `$baseURI` (inherited from the parent
class `Model`) can be accessed from outside the object. The remaining
ones: `$modelID` and `$dbConn` are private variables.
The former is the database internal identifier for the model. It is used to
avoid SQL joins on tables: *statements* and *models*, which results
in increased query performance. The latter is a reference pointer to the ADOConnection
object of the corresponding database object. The binding of a persisted model
to the specific database connection allows us to simultaneously work with various
models stored in multiple databases.

Note that RDF statements of a persisted model are not loaded into memory while
a `DbModel` is created. Most of the methods of this class are able
to query and manipulate the triples directly in the database, for example: `size()`,
`add()`, `remove()`, `contains()`, `containsAny()`,
`find()`, `rdqlQuery()`, `replace()`, `equals()`,
`addModel()`, etc. Delegating some tasks to the database management
system saves server resources and reduces the CPU usage. Only few functions
require all the statements to be loaded into memory for further processing.
These methods are, for instance: `saveAs()`, `unite()`,
`toStringIncludingTriples()`, `writeAsHtmlTable()`, `findRegex()`,
etc.

### Portability

One of the great advantages of RAP's database backend is the ability to support
multiple databases. On the other hand the portability has also its price. As
most database vendors practice product lock-in, the fastest way to query a certain
database is to use its proprietary extensions to SQL which are not standardized.
Moreover, even many widespread and very useful features like auto-incrementing
columns or binding variables in an SQL statement are not supported by all databases
(in this case the former has to be emulated by the method `_createUniqueModelID()`
of class `DbStore`, the latter unfortunately could not have been
implemented). All of this required writing portable SQL that would perform well
under all conditions.

Furthermore, handling of data types and indexes is also an area where differences
among various database vendors may occur. Therefore, RAP provides methods automatically
creating tables only for those two databases that have been tested - MsAccess
and MySQL. If you want to use other databases, you will have to create tables
manually according to RAP's abstract database
schema.

### Error and Transaction Handling

RAP's database backend offers an extensive error checking. In the case of public
methods all objects passed as parameters are checked whether or not they are
instances of the desired class. If not, an error message is shown on the screen.
Moreover, the user is informed if an error occurs while connecting to the database
or executing an SQL statement. Furthermore, transactions consisting of several
SQL statements, for instance, creating tables (`createTables()`),
adding (`add()`) or deleting an entire model (`delete()`),
are also being monitored for SQL errors. If an error occurs, the whole transaction
will be rolled back.

Additionally, the user is also given the possibility to turn the debugging
on, that will display all SQL statements before execution. To activate this
feature you have to edit the script `constants.php` and set the constant
`ADO_DEBUG_MODE` to "`1`", as shown below:

`define("ADODB_DEBUG_MODE", "1");`
